# Depression and self-care in diabetes; adjustment for misclassification bias: application of predictive weighting method

**DOI:** 10.1186/s12889-023-17412-x

**Published:** 2023-12-19

**Authors:** Zahra Kordi, Ahmad Khosravi, Akbar Fotouhi

**Affiliations:** 1 Department of Epidemiology and Biostatistics, School of Public Health, Tehran University of Medical Sciences, Tehran, Iran; 2 Vice-chancellery for Research, Shahroud University of Medical Sciences, Shahroud, Iran; 3 Department of Epidemiology, School of Public Health, Shahroud University of Medical Sciences, Shahroud, Iran

**Keywords:** Diabetes mellitus, Predictive value, Sensitivity and specificity, Depression

## Abstract

**Objective:**

This study aimed to investigate the association between depression and self-care in diabetic patients potentially influenced by biases in depression measurement using weighting the positive and negative predictive values.

**Methods:**

In this cross-sectional study, 1050 patients informedly consented to participate in the study. Using a WHO-5 well-being index, the participants were examined for depressive mood as exposure. The sensitivity and specificity of this index in a systematic review study were 0.86 and 0.81, respectively. Self-care (that is outcome) was assessed using the Summary of Diabetes Self-Care Activities (SDSCA) questionnaire. To correct the misclassification bias of exposure, the predictive weighting method was used in the multivariable logistic regression model adjusted for covariates. Bootstrap sample with replacement and simulation was used to deal with random error.

**Results:**

The mean age of patients was 42.8 ± 7.5 years. In this study, 70.1% of diabetic patients (*n* = 720) were depressed based on the questionnaire score and only 52.7% (*n* = 541) of them had appropriate self-care behaviors. Our study revealed a close relationship between self-care and covariates such as gender, depression, having comorbidities, abdominal obesity, economic status and education. The odds ratio of the association between depressive mood and lack of self-care in primary multivariable logistic regression was 2.21 (95% CI: 1.62-3.00, *p* < 0.001) and after misclassification bias adjusting, it was equal to 3.4 (95% CI: 1.7–6.6, *p* < 0.001). The OR percentage of bias was − 0.55.

**Conclusion:**

After adjusting for depression misclassification bias and random error, the observed association between depression and self-care was stronger. According to our findings, psychiatric interventions, and counseling and education along with self-care interventions are necessary for these patients. Special attention should be paid to male, low economic classes, less educated and those having a history of comorbidities along with psychological assessment when improving the care and progress of treatment in diabetic patients is expected. Future studies are needed to clarify the role of other psychological disorders on self-care of diabetics.

## Introduction

Diabetes is a metabolic disease that is estimated to affect about 693 million people worldwide by 2045, and the number of patients in Iran will increase from 4.5 million in 2017 to 9.2 million in 2030 [1, 2]. Therefore, prevention and control of diabetes are very important, and one of the main characteristics of control and prevention of diabetes is self-care of the disease, which include a range of activities such as blood sugar control, diet free from trans fatty acids, physical activity, daily checking of legs, taking medicine [3, 4]. Mindfulness practices can manage negative effects of depression, develop health-related behaviors, such as being physically active, and avoiding smoking and alcohol [5]. The results of a systematic review showed a poor adherence of the population to good self-care practices [6]. Self-care refers to the practice of health care, health management, and health promotion in individuals’ daily life [7]. The concept of self-care was assessed among people living with chronic health conditions [5, 7–9].

One of the most important risk factors that affect the self-care behaviors of diabetic patients is depression. As research shows, the prevalence of depression in diabetic patients is twice as high as in non-diabetics, and depression is considered an independent risk factor for the development of diabetes [10–12]. Studies conducted in different parts of the world, such as the one by Gonzalez and colleagues and the one by Richard Steven, indicate that 19% of diabetic patients have symptoms of major depression, and reducing depressive symptoms leads to increased patient self-care [3, 10]. A review study conducted in Iran estimated the prevalence of depression in diabetic patients to be 54.8% [13]. In another systematic review in Iran the prevalence of depression was reported as 61.8% (95% CI: 56.6–66.7) [14].

In epidemiological studies, depressive mood can be assessed by using various questionnaires such as BDI (Beck Depression Inventory), WHO-5 Well-Being Index, DASS-21 (Depression Anxiety Stress Scale-21) and self-rating depression scale (SDS) [15–18]. However, the ultimate definitive diagnosis is through the clinical interview [3]. One of the most concise and practical tools in the screening of depressive mood is the WHO-5 well-being index. A systematic review article on 18 different studies examined the psychological characteristics of this index for the diagnosis of depression. This review study reports the shortness, and high sensitivity, and high specificity (86% and 81%, respectively) of this index in the diagnosis of depressive mood as its advantages [16].

Therefore, due to the high prevalence of depression in diabetic patients and since various questionnaires with different diagnostic value (sensitivity and specificity less than 100%) are used to measure depressive mood, it is possible for misclassification to occurs in examining the association between depressive mood as the exposure and self-care as the outcome; misclassifications due to un-perfect tests [19]. Measurement error as the difference between the measured value of the variable and the actual value affects the validity of the study [20]. Measurement error and misclassification in two-by-two probability tables reduce the power of statistical inference to examine the association between the exposure and the outcome [21]. Moreover, errors in estimating standard errors (SE) decrease the accuracy of estimating depressive mood [22]. Various methods have been proposed to correct this error, one of which is the correction of exposure misclassification by using the sensitivity and the specificity of the questionnaire for the exposure variable (here depressive mood). The limitation of this method is that the apparent prevalence of exposure (depressive mood) in the study should not be more than the sensitivity of the test used, and it should not be less than the probability of false positive. This method is not suitable for correcting misclassification when the calculation method for the multivariate regression model is taken into account. To use this method, positive and negative predictive values can be calculated using sensitivity and specificity, and according to the definition of positive predictive value (i.e., the probability of real depression in people diagnosed with depression based on the questionnaire), this probability can be used as the actual exposure weight [19].

Therefore, to examine the association between exposure and outcome, the data weighting method through predictive value has been used in this study [19]. Using this method, the parameters under study can be estimated more accurately, so the purpose of this study is to correct this possible misclassification by weighing the positive and negative predictive values in the logistic regression model.

## Materials and methods

The present study is a cross-sectional study and the target population included all type 2 diabetic patients in the age range of 30 to 65 years who were registered as a diabetic in Diabetes Clinic of Shahroud, Charity Diabetes Center of Shahroud, and Healthcare Centers and were under medical care in these centers. After the initial assessing of the medical records of diabetic patients in each center, they have been contacted and asked to refer to the diabetes center or healthcare center. According to a systematic review in Iran [13], the prevalence of depression in diabetic patients was estimated 0.548 and 1226 samples is needed to estimate the true proportion within 4% with 95% confidence. Inclusion criteria included patients with type 2 diabetes, who speak Persian, and who had no severe diabetes complications. In this study, 1050 diabetic patients out of 1666 patients with active care accepted our invitation and participated in our study.

### Ethical considerations

This study was reviewed and registered in the Medical Ethics Committee of Tehran University of Medical Sciences with the code IR.TUMS.SPH.REC.1397.153 and was also reviewed by the ethical board of Shahroud University of Medical Sciences. Interviews with patients were conducted after obtaining the consent of the Vice Chancellor for Health of Shahroud University of Medical Sciences.

### Data collection

Patients’ data were measured and recorded by 2 full-time trained health experts and three part-time experts working in the centers. Participants were interviewed and clinically examined to record their demographic characteristics including (age, sex, occupation, marital status, years of education), having comorbidities (cardiovascular disease, chronic kidney disease and stroke), the economic status, duration of diabetes, cholesterol, systolic and diastolic blood pressure, height, weight, the last measured fasting blood sugar level, the time of fasting blood sugar measurement, and smoking status. Measurement of cholesterol, and fasting blood sugar along with other counseling services was free for patients if it was necessary.

To measure the economic status of the participants, 11 items of household amenities and individual properties (such as home and car ownership) were combined using the principal components analysis (PCA). According to the final scores, the participants in the study were assigned into three groups with high, moderate, and low economic status [23].

The blood pressure of each participant was measured twice in the right hand at a five-minute interval, and if the difference between the two measurements was more than 10 mm/Hg in systole or 5 mm/Hg in diastole, blood pressure was measured for the third time. The mean of the two measurements was recorded as the amount of systolic and diastolic pressure [24]. We generated a dichotomous variable in which a hypertensive patient is defined as an individual with blood pressure at least 140/90 mmHg or on antihypertensive medications. The cholesterol and FBS values were extracted from the patients’ medical records and the baseline was its measurement during the last three months, and if needed, the measurements for the patients were done. Cholesterol less than 200 mg/dL was considered normal [25].

For men, the waist circumference of 102 cm and above, and for women waist circumference of 88 cm and above were considered as abdominal obesity [26]. Body mass index was calculated by dividing weight in kilograms by height in meters to the power of two. Participants were divided into three groups: normal (BMI less than 25 kg/ m^2^), overweight (BMI 25 to 30 kg/ m^2^) and obese (BMI more than 30 kg/ m^2^) [27].

#### Summary of Diabetes Self-Care Activities (SDSCA)

This questionnaire is a valid 12-item self-report tool the new version of which rates five aspects (diet, exercise, blood glucose testing, foot care, and smoking) over the past seven days on a seven-point Likert scale which ranges from zero (no self-care during the last seven days) to 7 (self-care every seven days). One item is about smoking with a score of either seven or zero; if the patient smokes he gets a score of zero, and if he does not, he gets a score of 7. Higher scores indicate that the patient has had better self-care over the last seven days. In this study, we divided the level of self-care based on the total self-care score into two levels: poor (less than 50% of the score), and good i.e., people with a score above 50% [9, 28]. The construct validity of this questionnaire was evaluated using confirmatory factor analysis, and the SDSCA measure was found to be a valid one [29].

#### WHO-5 well-being index

Depressive mood in patients was assessed using the 5-item WHO-5 well-being index. This questionnaire is a short scale of 5 self-report questions and measures the level of positive mental health over the past two weeks on a 6-point Likert scale from zero (never) to 5 (always). Respondent’s raw score is theoretically in the range of zero (no mental health) to 25 (maximum mental health). This questionnaire has been used in various studies to measure depressive mood in different patients, including diabetics. A score of less than 13 indicates a depressive state and a score of 14 to 25 indicates good mental health. The validity and reliability of the WHO-5 well-being index has been confirmed in various studies [16, 30]. The internal consistency and convergent validity of Persian version of this questionnaire were evaluated in khosravi and et al. study [30]. WHO-5 has high clinimetric validity and it is a valid tool for screening of depressive mood [16].

### Data analysis

The data were analyzed using STATA v-14. The association between categorical variables was done using Chi-square test and comparison of two means was done using independent t-test. In this study, the association between predictors including depressive mood, hypertension, comorbidity, marital status, grouped age, duration of diabetes, BMI, abdominal obesity, education, economic status, smoking, cholesterol level with self-care was assessed using a multivariable logistic regression model once without considering misclassification bias of the depressive mood variable and once with adjustment misclassification of the depressive mood variable. In this model we entered all potential predictors from univariate analyses at a more relaxed Type I error rate (*p* < 0.25) [31]. For assessing the multicollinearity between variables we calculated the variance inflated factor (VIF) and degree of collinearity using tolerance index (1/vif) [32]. In our data the vif was equal 1.2 and tolerance index was 0.82. In this model, to adjust the exposure misclassification bias in the multivariable logistic regression model, the predictive value weighting method was used, which was explained in 2010 by Lyles and Lin [19]. The predictive value weighing (PVW) module was used to perform predictive value weighting for depressive mood misclassification in logistic regression. These predictive values (positive and negative) were used to run a weighted logistic regression model. Standard errors, p-values and confidence intervals are calculated using the bootstrap. For bias corrected estimates and random error we wrote a program with bootstrap sample with replacement. From the bootstrap sample (repeat = 200) we run our adjusted logistic regression model with predictive value weighting. The method used is as follows:

Using sensitivity (SE_y_= 0.86) and specificity (SP_y_= 0.81) of WHO-5 well-being index and apparent prevalence of exposure (depressive mood) based on study data (${\pi }_{Y}^{+})$, positive predictive value (PPV_y_) and negative predictive value (NPV_y_) are estimated at variable outcome levels (y = 0,1) using the solution of two linear equations (according to Formula 1), and given the definition of positive predictive value (probability of actual depressive mood in people diagnosed with depressive mood according to the questionnaire), this probability can be used as the weight of actual exposure in the logistic regression model (Formula 2) [19].

1 $$P_y=A_y^{-1}J$$

  Where:

$$P_y=\begin{pmatrix}{PPV}_y\\{NPV}_y\end{pmatrix},A_y\begin{pmatrix}\left({SE}_y-1\right)\pi_y^+\left[{SE}_y\left(\pi_y^+-1\right)^{-1}\right]&1\\1&\left({SP}_y-1\right)\left(\pi_y^+-1\right)\left(SP_y\pi_y^+\right)^{-1}\end{pmatrix}\text{and}\,J=\begin{pmatrix}1\\1\end{pmatrix}$$

2 $$\pi_y=PPV_y\pi_Y^+\;+\;\left(1-NPV_y\right)\left(1-\pi_y^+\right)(y=\text{0,1})$$

## Results

In this study, 656 women (63.9%) and 371 men (36.1%) were questioned and completed the self-care questionnaire (1027 patients of 1050). The mean ± standard deviation of the participants’ age was 42.8 ± 7.5 years. The narrative information of included sample in the analysis was shown in the Table 1. Most of them were older than 40 years (93.3%). The mean total WHO-5 score of our diabetics was 7.9 ± 7.2. In this study, 70.1% of diabetic patients (*n* = 720) had depressive mood (score less than 13) and 52.7% (*n* = 541) had appropriate self-care behaviors. The mean scores of self-care for men and women were 25.3 ± 11.7 and 29.7 ± 10.2, respectively, which shows a significant difference between the two sexes (t = 6.2, *p* < 0.001).

**Table 1:** Association between demographic variables and predictive factors with the level of self-care -univariate analysis

Variable	Self-care		Total (%)	χ^2^	*p*-value
	Good (%)	Poor (%)			
Age				9.97	0.002
Less than 40 years	49 (71.0)	20 (29.0)	69 (6.7)		
More than 40 years	492 (51.4)	466 (48.6)	958 (93.3)		
Sex				57.80	<0.001
Male	137 (36.9)	234 (63.1)	371 (36.1)		
Female	404 (61.6)	252 (38.4)	656 (63.9)		
Marital status				2.65	0.26
Single	5 (71.4)	2 (28.6)	7 (0.7)		
Married	457 (51.8)	426 (48.2)	883 (86.0)		
Widow/Divorced	79 (57.7)	58(42.3)	137 (13.3)		
The economic situation				43.25	<0.001
Low	219 (42.5)	296 (57.5)	515 (50.2)		
Moderate	155 (61.3)	98 (38.7)	253 (24.6)		
High	167 (64.5)	92 (35.5)	259 (25.2)		
Education					
Illiterate	94 (47.7)	103 (52.3)	197 (19.2)	12.72	0.01
Primary	207 (53.2)	182 (46.8)	389 (37.9)		
Diploma	194 (51.3)	184 (48.7)	378 (36.8)		
University	46 (73.0)	17 (27.0)	63 (6.1)		
Body mass index (BMI)				13.25	0.001
Normal	89 (58.6)	63 (41.4)	152 (14.8)		
Overweight	228 (46.7)	260 (53.3)	488 (47.5)		
Obesity	224 (57.9)	163 (42.1)	387 (37.7)		
Abdominal obesity				5.87	0.01
Normal	145 (46.9)	164 (53.1)	309 (30.1)		
Abnormal	396 (55.2)	322 (44.8)	718 (69.9)		
Comorbidity				19.17	<0.001
Yes	369 (48.6)	391 (51.4)	760 (74.0)		
No	170 (64.2)	95(35.8)	265 (25.8)		
Missing	2 (100.0)	0 (0)	2 (0.2)		
High blood pressure				3.87	0.05
Yes	199 (48.9)	208 (51.1)	407 (39.6)		
No	342 (55.2)	278 (44.8)	620 (60.4)		
Smoking					
Yes	12 (27.3)	32 (72.7)	44 (4.3)	11.90	<0.001
No	529 (53.8)	454 (46.2)	983 (95.7)		
Depressive mood					
Yes	329 (45.7)	391 (54.3)	720 (70.1)	47.12	<0.001
No	212 (69.1)	95 (30.9)	307 (29.1)		

In this study, the association between predictor variables and the level of self-care was assessed using univariate analysis and is presented in Table 1.

Table 2 shows the multivariate logistic regression model on the association between depressive mood and other predictor variables with self-care was assessed either without correction of misclassification or with correction of misclassification using the specificity and sensitivity of WHO-5 well-being index by inverse weighting predictive value method. In this model, the sensitivity and specificity of the questionnaire for measuring depressive mood were considered equal to 0.86 and 0.81, respectively, which were obtained based on the results of a systematic review article on 18 different studies [16]. The results of the multivariable logistic regression model showed that the male gender, the feeling of depression, having comorbidities, economic status, education and abdominal obesity are the main factors related to reducing the level of self-care. The odds ratio of the relationship between feeling depressed and lack of self-care is 2.21 (95% CI: 1.62-3.0, *p* = < 0.001) and with the correction of misclassification and random error, the odds ratio of feeling depressed and lack of self-care is equal to 3.4 (95% CI: 1.7–6.6, *p* = < 0.001). The OR percentage of bias was − 0.35 (that is: 2.21–3.4/3.4).

**Table 2:** Adjusted multivariable logistic regression with and without adjusting misclassification bias (sensitivity = 0.86 and specificity = 0.81) for depressive mood using a bootstrap sample replacement for calculation of simulation interval

Variables	Without adjusting misclassification bias			Adjusting misclassification bias		
	OR	95% CI	*p*-value	OR	95% CI	*p*-value
Depressive mood						
No	1	-		-	-	
Yes	2.21	1.62-3.00	<0.001	3.4	1.7- 6.6	<0.001
Sex						
Male	1	-		-	-	
Female	0.22	0.14 – 0.33	<0.001	0.21	0.13 – 0.35	<0.001
Comorbidity disease						
No	1	-		-	-	
Yes	1.58	1.14 – 2.19	0.006	1.51	1.03 – 2.22	0.03
Economic status						
Low	1	-		-	-	
Medium	0.54	0.38 – 0.75	0.001	0.61	0.42 – 0.89	0.01
High	0.58	0.40 – 0.83	0.003	0.94	0.52 – 1.17	0.24
Smoking						
No	1	-		-	-	
Yes	1.80	0.78 – 4.13	0.16	1.78	0.70 – 4.49	0.22
Education						
Illiterate	1	-		-	-	
Primary	0.75	0.51 - 1.10	0.15	0.71	0.47 – 1.05	0.09
Diploma	0.78	0.51 – 1.18	0.25	0.74	0.47 – 1.16	0.20
University	0.30	0.14 – 0.62	0.001	0.38	0.15 – 0.94	0.04
Age group						
Less than 40 years	1	-		-	-	
More than 40 years	1.57	0.87 – 2.83	0.11	1.44	0.76 – 2.70	0.25
Body mass index (BMI)						
Normal	1	-		-	-	
Overweight	1.26	0.82 – 1.93	0.28	1.29	0.79 – 2.100	0.30
Obesity	1.00	0.62 – 1.60	0.98	0.94	0.54 – 1.62	0.83
Abdominal obesity						
No	1	-		-	-	
Yes	2.03	1.30 – 3.18	0.002	2.07	1.26- 3.40	0.004
Hypertension						
No	1	-		-	-	
Yes	1.06	0.80 -1.41	0.66	1.03	0.76 – 1.40	0.82

## Discussion

In the study, 48.5% of patients had undesirable diabetes self-care behaviors and 70.1% of them felt depressed. According to the results of the present study, the odds of poor diabetic self-care in people who feel depressed are 2.21 as high as those who do not feel so. The adjusted OR in the misclassification adjusted model equal to 3.4 (95% CI: 1.7–6.6). The uncertainty model has shown this relationship to be larger. Depression affects the self-care behaviors of diabetic patients; depression increases the risk of poor glycemic control and complications of diabetes and reduces patients’ ability to perform self-care activities [33–35]. These findings are consistent with the findings of Siedes and et al. [10], Lin and et al. [36], and Gonzalez and et al. [3]. Other studies have shown that the risk of depression in diabetic patients is twice as high as the risk in the general population [37]. Moreover, the level of self-care in diabetic patients with depression is lower than that in other patients. Various studies have emphasized the importance of depression as a risk factor affecting the self-care behaviors of diabetic patients [36, 38–40]. In Ciechanowski’s study [41], the feeling of depression is associated with lower physical and mental functioning and reduced adherence to treatment and medication regimens, which is consistent with the results of our study.

In relation to proportion of undesirable diabetes self-care behaviors in our study (48.5% ), Bigdeli and et al. [42], Jordan [43], and Taghipour and et al. [12], also have reported undesirable diabetes self-care behaviors by the patients. Bigdeli and colleagues argued that the reason for the undesirable self-care behavior of patients in their study was the older age of the participants and the short duration of diabetes development. In their study, Sourani and et al. [44] reported low to moderate self-care ability of diabetic patients, which is consistent with the results of our study. Our study population characteristics may affect the self-care behaviors of the patients because obese people usually do not follow the treatment regimen and this reduces the level of self-care. In the present study, 70.1% of diabetic patients felt depressed and this finding is higher than the results in two systematic review studies (54.8% and 61.8%) [13, 14]. The prevalence of depressive mood among diabetic patients, for no good reason, is higher than that of other chronic medical conditions. However, factors such as pains caused by neuropathy, neuroendocrine mechanisms, pituitary-hypothalamic axis dysfunctions, and cerebral ischemia caused by vascular disorders are known as influencing factors [45]. Diabetic patients with high levels of depressive mood have reported poorer dietary status, a finding reported by Gonzalez and et al., and Seides [3, 10].

In the present study, there was not a significant relationship between age and diabetic self-care behavior in the misclassification adjusted exposure model, which was similar to the study of Bigdeli and et al. [42] Rather than age, self-care behaviors are more related to patients’ level of education and knowledge of the disease and the training they receive. In our study, females had better diabetic self-care behaviors than males and the odds ratio in the misclassification adjusted model after correction of exposure misclassification bias was 0.21 (95% CI: 0.13–0.35). The relationship between gender and self-care and the quality of life of diabetic patients has been reported by Long and et al. [46] and Chiu and et al. [47]. However, in the study of Bigdeli and et al., that relationship was not significant. In other studies, the self-care score did not differ significantly between the two genders [8, 48]. In addition to these contradictory results, it seems that females at all levels have higher self-care, which is due to their greater susceptibility to disease and more referrals for services. High-risk behaviors in males, such as smoking can be among other reasons for the lower level of self-care in men. The results of our study showed that smokers have poorer self-care. The odds ratio of smoking for poor diabetic self-care in the modified exposure model was not significant (OR = 1.78, 95% CI: 0.70–4.49). Gonzalez also has reported the effect of the smoking of diabetic patients on their depression [3]. As mentioned in the self-care questionnaire, smoking is an important factor in relation to lack of self-care.

The results of our study showed that people with moderate and high economic status had higher levels of diabetic self-care compared to people with low economic levels, and in the logistic regression model, moderate economic level compared to low economic level had a significant odds ratio of less than one. The results of the present study are consistent with the results of the study of Allahayari and et al. [49] and the study of Davari and et al. [50]. The main reason for this result could be more access to health services for people with higher economic status and also their high level of awareness.

### Limitation and strengths

The present study suffers from some limitations. First, it is a cross-sectional study. The main feature of cross-sectional studies is that data are collected over a period of time and this limits the ability to determine causal inference between variables due to causal reverse bias (i.e. relationship between obesity and diabetic self-care). Second, since participants self-reported their answers and some participants were illiterate therefore an interviewer needed to complete the questionnaire for them; precise answers might not have been provided for some questions. Also, unmeasured confounding, misclassification bias in covariates and outcomes could be affected on our estimates.Large sample size and careful assessment of diabetics are as strengths of our study. Other strengths of this study are using a new deterministic adjusting model for correction of misclassification bias and application of a multivariable logistic regression model.

## Conclusion

After adjusting for depression misclassification bias and random error using predictive value weighting and simulation, the observed association between feeling of depression and self-care was stronger. Using of this model for adjustment of misclassification bias can be recommended.

### Various

Finally, the results showed that felling of depression has a significant and strong relationship with self-care in diabetic patients that should be considered and followed in the planning and treatment of patients. According to our findings, psychiatric interventions, and counseling and education along with self-care interventions are necessary for these patients. More communication between the doctor and the patient, providing more services in the centers, and telephone follow-ups can be effective in increasing patients’ self-care. Special attention should be paid to male, low economic classes, less educated and those having a history of comorbidities along with psychological assessment when improving the care and progress of treatment in diabetic patients is expected. Future studies are needed to clarify the role of other psychological disorders on self-care of diabetics.

## Data Availability

Data are available through the request from the corresponding author.

## Abbreviations

SDSCA: Summary of Diabetes Self-Care Activities
WHO: World health organization
CI: Confidence interval
BDI: Beck Depression Inventory
DASS-21: Depression Anxiety Stress Scale-21
SE: Standard errors
PCA: Principal component analysis
SE_y_: Sensitivity
SP_y_: Specificity
NPVy: Negative predictive value
PPV_y_: Positive predictive value
VIF: Variance inflated factor
